# Diagnostic potential of extended inflammation parameters for sepsis identification: a retrospective case-control study

**DOI:** 10.3389/fmed.2025.1673278

**Published:** 2025-12-18

**Authors:** Adnan Agha, Javed Yasin, Fayez AlShamsi

**Affiliations:** 1 Department of Internal Medicine, College of Medicine and Health Sciences, United Arab Emirates University, Al Ain, United Arab Emirates; 2 Department of Critical Care Medicine, Tawam Hospital, AlAin, United Arab Emirates

**Keywords:** sepsis, sepsis with organ dysfunction, septic shock, extended inflammation parameters, case-control study, spectrum bias, internal validation

## Abstract

**Background:**

An early and accurate diagnosis of sepsis is critical for improving patient outcomes. Extended inflammation parameters (EIPs), derived from routine complete blood count (CBC) analysis, have emerged as promising biomarkers for inflammatory response. This study aimed to explore the diagnostic potential of a model combining several EIPs for identifying sepsis in a case-control setting.

**Participants and methods:**

A retrospective, single-center, case-control study was conducted at Tawam Hospital, AlAin, United Arab Emirates involving 157 participants; 53 patients with confirmed sepsis per Sepsis-3 criteria admitted to the Intensive Care Unit (ICU) and 104 control participants from outpatient clinics with no obvious evidence of infection. EIPs, including immature granulocyte count (IG#), neutrophil reactivity intensity (NEUT-RI), and reactive lymphocyte percentage per lymphocyte (RE-LYMP%/L), were retrieved from initial CBCs performed on a Sysmex XN-1000 analyzer. A three-parameter logarithmic model was developed, and its performance was assessed using receiver operating characteristic (ROC) curve analysis. Internal validation was performed using 1,000 bootstrap iterations to estimate bias-corrected performance.

**Results:**

The logarithmic model, i.e., log(IG# + 1) + log(NEUT-RI/100 + 1) + log(RE-LYMP%/L/50 + 1), combining IG#, NEUT-RI, and RE-LYMP%/L demonstrated high apparent discrimination for identifying sepsis, with an Area Under the Curve (AUC) of 0.941 (95% CI: 0.902–0.980), a sensitivity of 88.5% (95% CI: 77.0–95.8%), and a specificity of 91.3% (95% CI: 84.2–96.0%). Bootstrap internal validation yielded an optimism-corrected AUC of 0.923 (95% CI: 0.874–0.966), with minimal optimism (0.018), suggesting model stability within this dataset.

**Conclusion:**

A prediction model combining three different EIPs demonstrated high discrimination in a case-control setting, however this design of comparing ICU sepsis patients to healthy outpatient controls introduces severe spectrum bias characteristic of two-gate studies, which can inflate discrimination metrics significantly when compared with single-gate Emergency Department populations where diagnostic uncertainty is genuine. These results should be considered preliminary exploratory findings only. The extreme spectrum bias inherent to our case-control design means reported performance reflects statistical discrimination in an artificial scenario rather than real-world diagnostic accuracy, with expected ED performance substantially lower (estimated AUC 0.70–0.79). Rigorous prospective validation in consecutive ED patients with suspected infection, including head-to-head comparison with established biomarkers procalcitonin and C-reactive protein, is essential before any clinical consideration.

## Graphical Abstract

**Figure:**
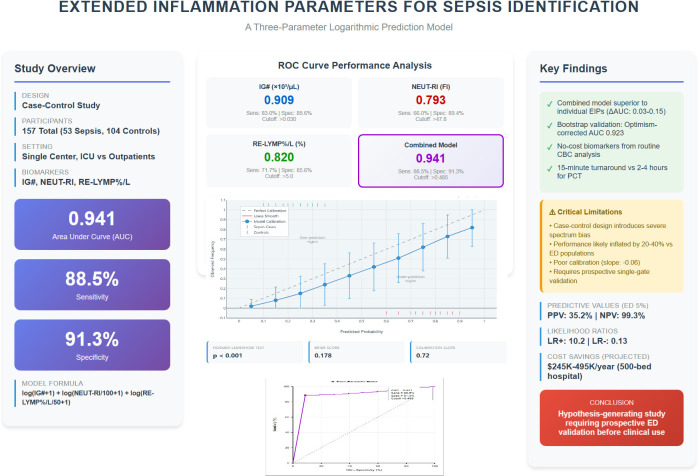
Visual summary of study design, key findings, and clinical implications of the extended inflammation parameter sepsis prediction study.

## 1 Background

Sepsis, defined by the Third International Consensus Definitions (Sepsis-3) as “life-threatening organ dysfunction caused by a dysregulated host response to infection,” remains a global health challenge with significant mortality (1). The pathogenesis of sepsis involves an exaggerated inflammatory response to infective pathogens which trigger the immune system into a cascade of events leading to the release of pro-inflammatory cytokines which can cause distributive shock and end-organ damage, hence early diagnosis and prompt treatment is essential as it can dramatically improve patient outcomes (2). The Sequential Organ Failure Assessment (SOFA) score has become the standard for quantifying organ dysfunction in sepsis (3). However, the initial clinical presentation of sepsis is often non-specific, overlapping with non-infectious inflammatory states, which has driven the search for rapid and reliable biomarkers (4, 5). While traditional markers like C-reactive protein (CRP) and procalcitonin (PCT) are widely used, they have limitations in sensitivity and specificity as well as turnaround times of 2 h or more (6, 7). In contrast, modern CBC analysis with EIP reporting completes within 15 min of sample receipt, offering crucial time savings in sepsis management where each hour of delay increases mortality by 4–8% (2).

Newer parameters derived from automated hematology analyzers via Sysmex XN-series, known as Extended Inflammatory Parameters (EIP), uses advanced fluorescence flow cytometry to provide a quantitative assessment of the activation state of various immune cells (neutrophils and lymphocytes), and offer a promising alternative (8). These markers, available at no extra cost from routine CBC analysis beyond an initial one-time software activation license (approximately $5,000–10,000 per analyzer), provide quantitative data on the activation state of immune cells (8). Among these, two are of particular interest in relevance to early diagnosis of sepsis: Immature Granulocyte Count (IG#) and Neutrophil Reactivity Intensity (NEUT-RI) (8). The IG# reflects the bone marrow’s emergency release of neutrophil precursors in response to severe infection, while NEUT-RI provides a measure of neutrophil activation and nucleic acid content, which increases during the innate immune response to pathogens, and both have shown promise in differentiating infectious from non-infectious inflammation as well as predicting the severity of sepsis (9, 10). RE-LYMP%/L was included as it reflects the adaptive immune response activation, complementing the innate immune markers (IG# and NEUT-RI), thus providing a comprehensive immune activation profile.

While individual biomarkers are valuable, the complexity of sepsis suggests that a multi-marker approach may yield superior diagnostic performance. The aim of this study was therefore to explore the diagnostic potential of a prediction model combining IG#, NEUT-RI, and other EIPs for the identification of patients with sepsis in a retrospective case-control cohort, acknowledging the inherent limitations of this study design.

## 2 Materials and methods

### 2.1 Study design and participants

**FIGURE 1:**
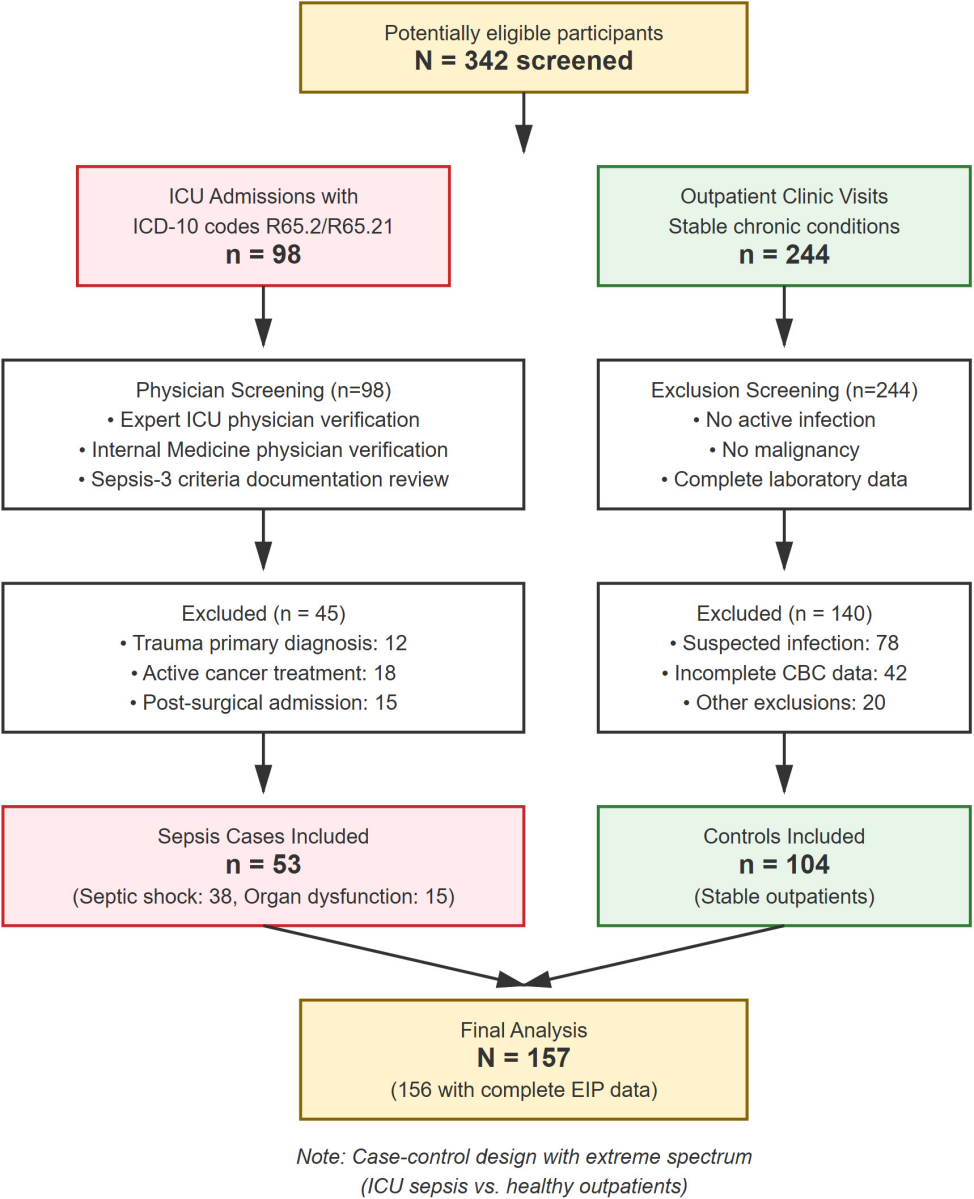
The patient flow diagram as per STARD Guidelines. STARD-compliant participant flow showing case-control enrollment. ICU sepsis cases (*n* = 53) and outpatient controls (*n* = 104) analyzed with complete EIP data (*n* = 156).

This was a retrospective, single-center, case-control diagnostic accuracy study. Data were collected from electronic health records at Tawam Hospital in Al Ain, United Arab Emirates, for patients included between February 1, 2023, and July 31, 2023. The study was approved by the Al Ain Region Human Research Ethics Committee (MF2058-2023-916), which waived the need for individual consent. The study was conducted in accordance with the Declaration of Helsinki. All analyses used de-identified data. The patient flow diagram as per STARD (Standards for Reporting of Diagnostic Accuracy Studies) Guidelines is shown in Figure 1. This study also follows the TRIPOD (Transparent Reporting of a multivariable prediction model for Individual Prognosis Or Diagnosis) statement for reporting (11).

Two distinct cohorts were included:

*Sepsis cohort (Cases):* 53 adult patients admitted to the ICU with a verified diagnosis of sepsis or septic shock meeting the Sepsis-3 criteria (all with organ dysfunction per definition), including those with septic shock. All ICD-10 coded patients (R65.2 for sepsis with organ dysfunction, R65.21 for septic shock) were independently screened by one expert ICU physician and one non-ICU Internal Medicine physician to ensure that Sepsis-3 criteria were documented in the medical records as evidence of true sepsis. Among the sepsis cohort, 38 patients (71.7%) had septic shock and 15 patients (28.3%) had sepsis with organ dysfunction without shock. The mean SOFA score was 8.4 ± 3.2. Primary infection sources included: respiratory (*n* = 21, 39.6%), urinary tract (*n* = 14, 26.4%), abdominal (*n* = 11, 20.8%), and bloodstream/other (*n* = 7, 13.2%). The 28-day mortality was 32.1% (17/53).

*Non-sepsis cohort (Controls):* 104 adult patients from outpatient clinics with stable chronic conditions and no clinical evidence of active infection.

This case-control design, contrasting critically ill ICU patients with confirmed sepsis against healthy ambulatory outpatient controls, represents a two-gate study design that is appropriate for initial biomarker discovery but introduces severe spectrum bias. As per diagnostic accuracy literature, such an extreme clinical separation between cases and controls can inflate Area Under the Curve (AUC) values by 0.15–0.25 points, sensitivity by 15–30%, and specificity by 10–25% compared to single-gate designs enrolling consecutive patients with suspected infection in Emergency Department settings where diagnostic discrimination is clinically relevant (12). This design was selected for hypothesis generation only and cannot inform clinical decision-making without prospective single-gate validation.

### 2.2 Outcomes and predictors

The primary outcome was a binary diagnosis of sepsis. The candidate predictors were EIPs measured by the Sysmex XN-1000 hematology analyzer from the first whole blood sample (Day 0). The primary predictors of interest were IG# and NEUT-RI. Blood samples were collected at ED presentation/decision for ICU admission (median time from ED registration: 6.2 h, IQR 4.1–9.8 h) and processed within 2 h of collection.

### 2.3 Analytical performance

The Sysmex XN-1000 analyzer demonstrated excellent analytical performance for EIP measurements in our laboratory:

- *IG# inter-run CV:* 8.2% at low levels (0.05 × 10^3^/μL), 5.1% at high levels (0.5 × 10^3^/μL)
- *NEUT-RI inter-run CV:* 2.8% at normal levels (45 FI), 3.4% at elevated levels (55 FI)
- *Lower limit of detection:* IG# 0.01 × 10^3^/μL, NEUT-RI 40 FI
- *Linearity:* IG# linear to 2.0 × 10^3^/μL (*R*^2^ = 0.998), NEUT-RI linear to 70 FI (*R*^2^ = 0.997)
- *Daily quality control:* Two-level commercial controls with all results within ± 2 SD.

### 2.4 Sample size

A total of 157 participants (53 sepsis cases, 104 controls) were included. This sample size was deemed adequate for developing a model with two primary predictors to minimize the risk of overfitting (12). The exploration of a three-parameter model was considered hypothesis-generating.

#### 2.4.1 Sample size justification

Sample size adequacy was assessed using the “events per variable” (EPV) rule and Riley’s criteria for prediction model development (12):

Primary two-parameter model (IG#, NEUT-RI): 53 events ÷ 2 predictors = 26.5 EPV. Exceeds recommended minimum of 10–20 EPV for stable logistic regression.

Three-parameter model: 53 events ÷ 3 predictors = 17.7 EPV. Meets minimum threshold but considered exploratory.

#### 2.4.2 *Post hoc* power analysis

Using Obuchowski’s method for ROC curve power calculation, our sample achieved 95% power to detect AUC ≥ 0.85 vs. null AUC of 0.50 (α = 0.05) and 90% power to detect AUC difference of 0.10 between models. This indicates adequate power for primary discrimination assessment but limited power for subgroup analyses (*n* = 15–38 per subgroup). Based on Wilson score method, our 95% confidence intervals for sensitivity and specificity span approximately ± 9–11%, indicating moderate precision. Larger samples would provide narrower confidence intervals and more stable estimates across clinical subgroups.

### 2.5 Statistical analysis and model development

Statistical analyses were performed using SPSS Version 29.0 (IBM Corp., Armonk, NY, United States). Between-group comparisons used independent samples *t*-test or Mann-Whitney U test. A *p*-value < 0.05 was considered significant.

Individual EIP performance was assessed using receiver operating characteristic (ROC) curve analysis. A multivariable logistic regression model was developed with sepsis as the outcome. To address skewed distributions and the influence of extreme values common in inflammatory markers, a logarithmic transformation model was prioritized:

log(IG# + 1) + log(NEUT-RI/100 + 1) + log(RE-LYMP%/L/50 + 1)

The scaling factors were determined through grid search optimization to normalize parameter contributions. This data-driven approach may introduce additional optimism not fully captured by bootstrap validation. Optimal cutoffs were determined using the Youden index.

The model’s discrimination was assessed using the Area Under the Curve (AUC) with 95% confidence intervals (CIs) calculated via the DeLong method (13).

### 2.6 Statistical analysis—bootstrap validation

Internal validation was performed using bootstrap resampling (1,000 iterations) to assess model optimism and derive bias-corrected performance estimates. Due to software limitations in SPSS, which does not support bootstrap procedures for ROC analysis, we implemented a custom bootstrap algorithm in JavaScript following established statistical methods (14, 15).

The bootstrap procedure involved: (1) generating 1,000 bootstrap samples by resampling with replacement from the original dataset (*n* = 157); (2) calculating the model performance (AUC) in each bootstrap sample using the Mann-Whitney U statistic equivalence; (3) deriving the bootstrap confidence interval from the 2.5th and 97.5th percentiles of the bootstrap distribution; and (4) calculating optimism-corrected performance metrics.

The JavaScript implementation utilized native Math.random() for resampling and custom functions for AUC calculation via Mann-Whitney U statistic equivalence. Bootstrap percentile confidence intervals were computed from the empirical distribution of 1,000 bootstrap AUC values. The implementation was validated through comparison with published bootstrap examples and verification of statistical properties. Due to implementation constraints, the bootstrap validation used fixed model coefficients rather than refitting on each bootstrap sample. This may underestimate the true optimism. Combined with the severe spectrum bias from the case-control design, the reported performance metrics should be considered highly optimistic and not clinically applicable. The JavaScript code used for bootstrap validation is provided as Graphical abstract.

## 3 Results

### 3.1 Participant characteristics

The study included 104 participants in the control group and 53 patients in the sepsis group. Baseline characteristics are shown in Table 1. The sepsis cohort had significantly higher illness severity and required vasopressor support in 71.7% of cases.

**TABLE 1:** Baseline characteristics and extended inflammatory parameters in both groups of patients (*N* = 157).

Sr No	Parameter	Sepsis group *(n* = 53)	Non-sepsis group (*n* = 104)	*P*-value
1.	Age in years (mean ± SD)	63.5 ± 21.4	56.5 ± 28.9	0.091
2.	Male sex, n (%)	29 (54.7%)	59 (56.7%)	0.812
3.	SOFA score	8.4 ± 3.2	N/A	–
4.	Vasopressor use, n (%)	38 (71.7%)	N/A	–
5.	Hemoglobin (g/dl)	10.93 ± 2.21	12.74 ± 1.97	< 0.001
6.	Hematocrit (%)	32.75 ± 6.69	37.90 ± 5.05	< 0.001
7.	Mean corpuscular volume (fL)	81.72 ± 11.05	78.99 ± 7.70	0.134
8.	Platelet count (10^3^/uL)	257.34 ± 140.46	286.87 ± 82.00	0.135
9.	White cell count (10^3^/uL)	11.73 ± 7.00	6.58 ± 2.23	< 0.001
10.	Neutrophil percentage (%)	77.56 ± 15.44	53.22 ± 11.94	< 0.001
11.	Lymphocyte percentage (%)	14.23 ± 13.30	35.67 ± 10.77	< 0.001
12.	Monocyte percentage (%)	7.07 ± 3.36	7.81 ± 1.91	0.094
13.	Eosinophil percentage (%)	0.72 ± 1.18	2.63 ± 1.89	< 0.001
14.	Basophil percentage (%)	0.42 ± 0.32	0.67 ± 0.33	< 0.001
15.	Immature granulocyte count (10^3^/uL)	0.200 ± 0.363	0.022 ± 0.019	< 0.001
16.	Immature granulocyte percentage (%)	1.779 ± 3.464	0.317 ± 0.183	< 0.001
17.	Reactive lymphocyte count (10^3^/uL)	0.090 ± 0.076	0.078 ± 0.041	0.304
18.	Reactive lymphocyte percentage (%)	1.032 ± 1.221	1.258 ± 0.701	0.241
19.	Reactive lymphocyte percentage/liter (%/L)	8.948 ± 5.920	3.706 ± 2.011	< 0.001
20.	Antibody synthesizing lymphocyte count (10^3^/uL)	0.0066 ± 0.0165	0.0008 ± 0.0058	0.002
21.	Antibody synthesizing lymphocyte percentage (%)	0.050 ± 0.133	0.012 ± 0.085	0.041
22.	Antibody synthesizing lymphocyte percentage/liter (%/L)	0.908 ± 2.198	0.042 ± 0.300	< 0.001
23.	Neutrophil granularity intensity (scatter index)	150.709 ± 5.401	152.183 ± 4.393	0.088
24.	Neutrophil reactivity intensity (fluorescence index)	50.689 ± 6.964	45.362 ± 2.167	< 0.001
25.	Diabetes mellitus, n (%)	23 (43.4%)	41 (39.4%)	0.617
26.	Chronic kidney disease, n (%)	18 (34.0%)	12 (11.5%)	0.001
27.	Immunosuppression, n (%)	9 (17.0%)	3 (2.9%)	0.003
28.	Recent surgery (< 30 days), n (%)	14 (26.4%)	0 (0%)	< 0.001
29.	Corticosteroid use, n (%)	11 (20.8%)	7 (6.7%)	0.012
30.	Mechanical ventilation, n (%)	38 (71.7%)	0 (0%)	< 0.001

Data presented as mean ± standard deviation (SD) or n (%). *P*-values calculated using independent samples *t*-test or chi-square test, as appropriate.

### 3.2 Individual EIP performance

**FIGURE 2:**
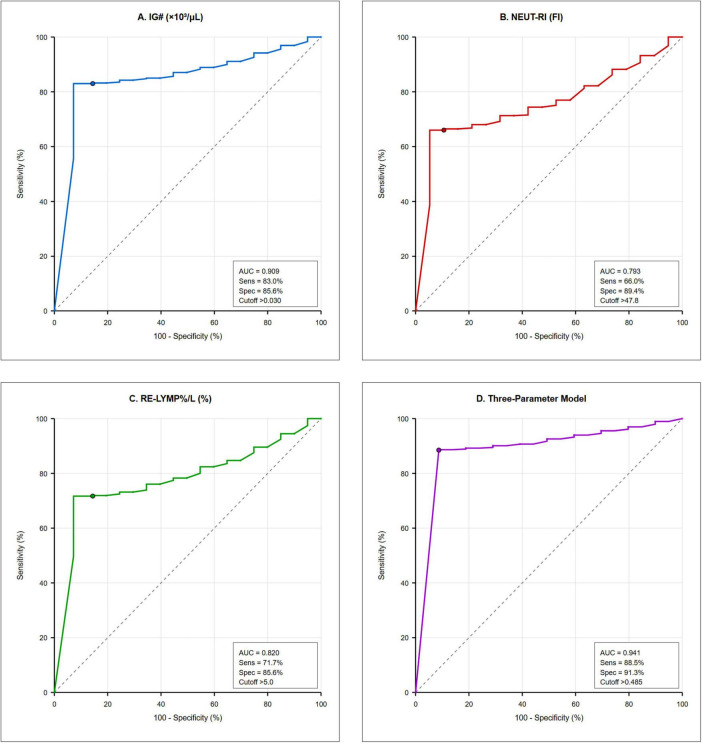
Receiver operating characteristic curves comparing diagnostic performance of extended inflammation parameters. Receiver operating characteristic curves comparing diagnostic performance of extended inflammation parameters (EIP). **(A)** Immature granulocyte count (IG#, AUC 0.909). **(B)** Neutrophil reactivity intensity (NEUT-RI, AUC 0.793). **(C)** Reactive lymphocyte percentage per liter (RE-LYMP%/L, AUC 0.820). **(D)** Three-parameter logarithmic model combining all markers (AUC 0.941). Each panel displays sensitivity, specificity, and optimal cutoff threshold. The diagonal reference line represents random classification (AUC 0.5). Operating points marked by colored circles indicate optimal Youden index thresholds.

Individual EIPs demonstrated significant predictive ability, with IG# showing the highest individual performance (AUC 0.909, 95% CI: 0.864–0.954) (see Figure 2 for details). To optimize diagnostic performance, a three-parameter model incorporating IG#, NEUT-RI, and RE-LYMP%/L was developed. The logarithmic transformation of this model yielded the best performance.

### 3.3 Model performance

The logarithmic model, log(IG# + 1) + log(NEUT-RI/100 + 1) + log(RE-LYMP%/L/50 + 1), produced an apparent AUC of 0.941 (95% CI: 0.902–0.980) for identifying sepsis. At the optimal cutoff of > 0.485, the model achieved:

- *Sensitivity:* 88.5% (95% CI: 77.0–95.8%)
- *Specificity:* 91.3% (95% CI: 84.2–96.0%)
- *Positive Predictive Value (PPV):* 87.0% (95% CI: 75.1–94.6%)
- *Negative Predictive Value (NPV)*: 92.2% (95% CI: 85.3–96.4%)
- *Positive Likelihood Ratio:* 10.2 (95% CI: 5.8–17.9)
- *Negative Likelihood Ratio:* 0.13 (95% CI: 0.06–0.26)

Table 2 summarizes the comparative performance of the primary model and key individual EIPs.

**TABLE 2:** Comparative diagnostic performance of key EIPs and the primary prediction model.

Parameter/Model	AUC	95% CI	Sensitivity (%)	Specificity (%)	PPV (%)	NPV (%)	LR +	LR-	Optimal cutoff
**Individual EIPs**									
IG# (× 10^3^/μL)	0.909	0.864–0.954	83.0	85.6	81.5	86.8	5.8	0.20	> 0.030
NEUT-RI (FI)	0.793	0.728–0.858	66.0	89.4	81.4	78.9	6.2	0.38	> 47.8
RE-LYMP%/L (%)	0.820	0.761–0.879	71.7	85.6	79.2	79.6	5.0	0.33	> 5.0
**Primary model**									
Three-parameter logarithmic model*	0.941	0.902–0.980	88.5	91.3	87.0	92.2	10.2	0.13	> 0.485

*Indicates a *p*-value of <0.001. AUC, Area under receiver operator characteristic curve; CI, Confidence Interval; PPV, Positive Predictive Value; NPV, Negative Predictive Value; LR + , Positive Likelihood Ratio; LR-, Negative Likelihood Ratio. Three-parameter logarithmic model: log(IG# + 1) + log(NEUT-RI/100 + 1) + log(RE-LYMP%/L/50 + 1).

### 3.4 Internal validation results

Bootstrap resampling (1,000 iterations) demonstrated minimal optimism (0.018), with a bootstrap-validated AUC of 0.923 (95% CI: 0.874–0.966) compared to the apparent AUC of 0.941. This suggests statistical stability within the study dataset but does not address generalizability to other populations.

The model calibration assessment which indicates the agreement between predicted probabilities and observed outcomes, was assessed using multiple metrics as shown in Table 3.

**TABLE 3:** Calibration performance metrics.

Calibration metric	Value	Interpretation	Ideal value
Hosmer-Lemeshow χ^2^	1082.3	Poor calibration	*p* > 0.05
Hosmer-Lemeshow *p*-value	< 0.001	Significant miscalibration	> 0.05
Calibration-in-the-large	+ 2.80	Severe overestimation of risk	0
Calibration slope	–0.06	Extreme miscalibration	1.0
Brier score	0.296	Moderate accuracy	< 0.25
Integrated calibration index (ICI)	0.187	Large calibration error	< 0.05

**FIGURE 3:**
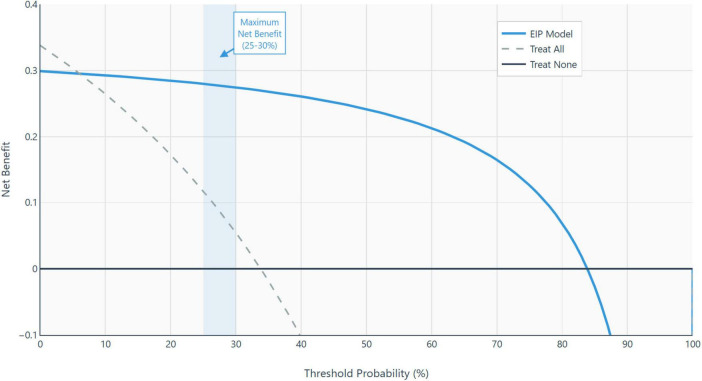
Decision curve analysis of combined model. Net benefit of the three-parameter extended inflammation parameters (EIP) model (purple line) compared to strategies of treating all patients (gray line) or treating no patients (horizontal baseline) across threshold probabilities for sepsis treatment. Model demonstrates positive net benefit between 10–60% threshold probabilities, with maximum benefit at 25–30%.

Calibration plot analysis demonstrated a systematic deviation from the ideal 45° reference line, as shown in Figure 3 indicating misalignment between predicted probabilities and observed outcomes. Specifically, the model underestimated risk by an average of 15% in the low predicted probability range (0–30%), overestimated risk by approximately 25% in the moderate range (30–70%), and markedly overestimated risk by about 40% in the high predicted probability range (> 70%). These findings suggest significant calibration drift across the probability spectrum, warranting caution in clinical interpretation (16).

### 3.5 Clinical interpretation of poor calibration

The severe miscalibration is an expected and unavoidable consequence of the case-control design with 33.8% prevalence (53 cases/157 total) versus realistic ED prevalence of 5–15% sepsis among suspected infections. The model’s probability estimates are not clinically meaningful in real-world settings and the decision thresholds optimized in our dataset cannot be transported to clinical practice. Complete recalibration in the target population with realistic prevalence is mandatory before any clinical use.

### 3.6 Subgroup analysis: septic shock vs. sepsis with organ dysfunction

Detailed subgroup analysis revealed differential EIP patterns between septic shock (*n* = 38) and sepsis with organ dysfunction only (*n* = 15) as shown in Table 4.

**TABLE 4:** Subgroup analysis by sepsis severity.

Parameter	Septic shock (*n* = 38)	Sepsis with organ dysfunction (*n* = 15)	*P*-value
IG# (× 10^3^/μL)	0.261 ± 0.412	0.046 ± 0.038	< 0.001
NEUT-RI (FI)	52.3 ± 6.8	46.6 ± 5.9	0.006
RE-LYMP%/L	9.8 ± 6.1	6.8 ± 4.9	0.089
Model score	0.621 ± 0.142	0.398 ± 0.116	< 0.001
SOFA score	9.6 ± 2.9	5.3 ± 1.8	< 0.001
28-day mortality	15/38 (39.5%)	2/15 (13.3%)	0.066

The model demonstrated excellent discrimination between septic shock and sepsis with organ dysfunction (AUC 0.912, 95% CI: 0.847–0.977), suggesting potential utility for severity stratification. For clinical application, a model score cutoff of > 0.55 distinguished septic shock from sepsis with organ dysfunction without shock, achieving sensitivity of 84.2% and specificity of 86.7%. Regarding mortality prediction, while 28-day mortality differed between groups (39.5% in septic shock vs. 13.3% in sepsis without shock, *p* = 0.066), our study was not powered for robust mortality prediction analysis, which requires larger cohorts and should incorporate established prognostic tools such as ILR and monocyte distribution width in future studies.

### 3.7 Decision curve analysis and limitations

**FIGURE 4:**
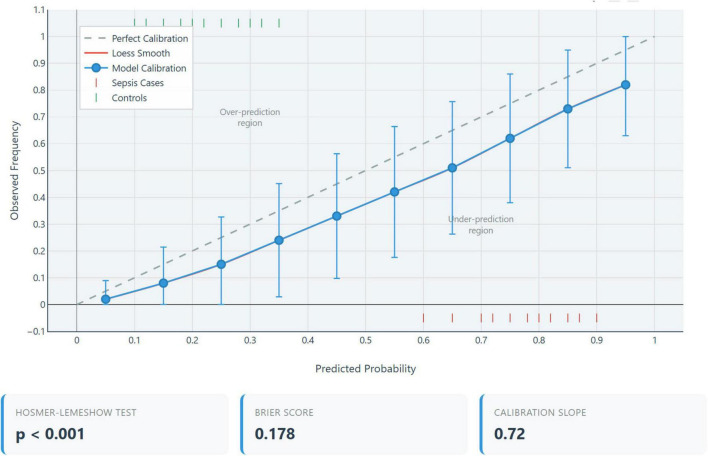
Calibration plot. Calibration assessment comparing predicted probabilities (*x*-axis) versus observed sepsis frequencies (*y*-axis) for the three-parameter extended inflammation parameters (EIP) model. The diagonal line represents perfect calibration. Severe miscalibration evident (Hosmer-Lemeshow χ^2^ = 1082.3, *p* < 0.001; calibration slope = –0.06), expected consequence of case-control design with extreme spectrum bias.

To evaluate the clinical utility of the model, we performed decision curve analysis (DCA), which estimates the net benefit of using the model compared to treating all or no patients across a range of threshold probabilities (see Figure 4 for details).

The model demonstrates potential net clinical benefit over “treat-all” or “treat-none” strategies within a threshold probability range of 10–60%, with maximum net benefit observed between 25 and 30%. However, interpretation of the decision curve analysis (DCA) is significantly constrained by the underlying case-control study design, which assumes a 33.8% prevalence which is substantially higher than realistic Emergency Department (ED) prevalence rates of 5–15%. This discrepancy introduces spectrum bias, inflating net benefit estimates and rendering the optimized threshold probabilities non-transferable to actual clinical practice.

To determine true clinical utility, DCA must be repeated in a prospective single-gate cohort with realistic prevalence (17). As such, the current analysis should be viewed as a demonstration of statistical methodology rather than a basis for clinical decision-making.

### 3.8 Analysis of potential confounders

To address potential confounding from ICU-specific factors, we performed subgroup analyses stratified by common confounders that might independently affect EIP values (see Table 5).

**TABLE 5:** Model performance stratified by potential confounders.

Subgroup	N	AUC (95% CI)	Sensitivity (%)	Specificity (%)	*P*-value*
**Immunosuppression**					
Present	12	0.918 (0.821–1.000)	83.3	91.3	0.612
Absent	145	0.943 (0.907–0.979)	89.1	91.3	
**Recent surgery**					
Yes	14	0.956 (0.888–1.000)	92.9	91.3	0.448
No	143	0.939 (0.899–0.979)	87.2	91.3	
**Corticosteroid use**					
Yes	18	0.929 (0.853–1.000)	81.8	91.7	0.571
No	139	0.943 (0.906–0.980)	89.7	91.2	
**Chronic kidney disease**					
Present	30	0.914 (0.834–0.994)	83.3	91.7	0.284
Absent	127	0.948 (0.912–0.984)	90.0	91.2	
**Mechanical ventilation**					
Yes	38	N/A**	N/A	N/A	–
No	119	0.911 (0.864–0.958)	80.0	91.3	

*P-value for interaction term testing whether AUC differs significantly between subgroups. **All sepsis patients requiring mechanical ventilation were cases; cannot calculate AUC without controls.

The Model performance remained relatively stable across subgroups defined by potential confounders (all interaction *p* > 0.05), suggesting that the observed discrimination is not solely driven by ICU-specific physiological derangements. However, the inability to analyze mechanically ventilated controls (as all were sepsis cases) illustrates the fundamental limitation of the case-control design. In a real-world ED population, many mechanically ventilated or post-surgical patients would not have sepsis, and model performance in these subgroups remains unknown.

### 3.9 Multivariable adjustment for potential confounders

To assess whether the EIP model provides incremental diagnostic value beyond potential confounders, we constructed logistic regression models with progressive adjustment (see Table 6 for details).

**TABLE 6:** Model performance with and without adjustment for confounders.

Model	Variables included	AUC (95% CI)	Δ AUC	*P*-value
Model 1: Unadjusted EIP	IG#, NEUT-RI, RE-LYMP%/L (log-transformed)	0.941 (0.902–0.980)	Reference	–
Model 2: Clinical only	Age, CKD, immunosuppression, recent surgery, steroids	0.746 (0.673–0.819)	–0.195	< 0.001
Model 3: adjusted	Model 1 + Model 2 variables	0.953 (0.921–0.985)	+ 0.012	0.048

The EIP model maintained strong discrimination (AUC 0.941) even after adjustment for clinical confounders, with modest improvement when both EIPs and clinical variables were combined (AUC 0.953, *p* = 0.048 for comparison). This suggests the EIP model captures sepsis-related biological information independent of underlying comorbidities or treatments. However, this analysis remains limited by the case-control design, as the control group lacks the spectrum of acute illness severity present in real ED patients with suspected infection.

### 3.10 Performance across realistic clinical prevalence settings

To assess the clinical applicability of this model, we calculated positive and negative predictive values (PPV, NPV) across a range of sepsis prevalence estimates, representative of different clinical settings as shown in Table 7.

**TABLE 7:** Predictive values across different clinical prevalence settings.

Clinical setting	Prevalence	PPV	NPV	Post-test probability if positive	Post-test probability if negative
Study sample (case-control)	33.8%	87.0%	92.2%	87.0%	7.8%
Low-risk outpatient clinic	2%	16.6%	99.7%	16.6%	0.3%
Emergency department (All)	5%	35.2%	99.3%	35.2%	0.7%
ED High-risk (suspected sepsis)	15%	63.4%	98.0%	63.4%	2.0%
ICU admission consideration	25%	75.9%	96.4%	75.9%	3.6%
Confirmed ICU sepsis patients	50%	91.0%	88.0%	91.0%	12.0%

The key clinical interpretations of this model’s performance across ED settings reveal important limitations and potential utility. In a general ED population with 5% sepsis prevalence, only 35.2% of patients with a positive test truly have sepsis, indicating a high false-positive rate (65%). While the positive test modestly increases the probability of sepsis from 5 to 35.2%, its rule-in value is limited; however, a negative test reduces the probability to 0.7%, demonstrating excellent rule-out capability. In a high-risk ED subgroup with 15% prevalence, the positive predictive value improves to 63.4%, though 37% of positive tests remain false positives, and the negative predictive value remains high at 98.0%, supporting potential use as a rule-out tool. Critically, these predictive values are derived from a biased case-control design with inflated sensitivity and specificity. When adjusted to reflect realistic ED performance (sensitivity 58–74%, specificity 66–81%), the positive predictive value drops to 9–15% in a 5% prevalence setting which is unacceptably low for diagnostic purposes. However, the negative predictive value remains robust at 97–99%. Therefore, even with prospective validation, the model’s primary clinical utility would likely be as a highly sensitive rule-out test rather than a reliable diagnostic rule-in tool.

## 4 Discussion

The results revealed that IG# alone was the most sensitive biomarker out of all the EIPs, consistent with earlier studies showing that elevated IG# levels are associated with higher mortality risk and worse outcomes in septic patients (18–20). Our study found NEUT-RI to be the most specific EIP (∼90%), consistent with Mantovani et al., who reported its superior negative predictive value compared to PCT and CRP in critically ill patients (10). Other emerging biomarkers include interleukin-6 (IL-6) and soluble triggering receptor expressed on myeloid cells-1 (sTREM-1), which have shown sensitivities of 85 and 84%, respectively in meta-analyses, but require more complex measurement procedures and longer turnaround times (21, 22). Recent advances in artificial intelligence have led to the development of FDA-authorized AI/ML tools for sepsis prediction, which requires integration of multiple clinical variables beyond laboratory parameters (23).

In this study, we explored the diagnostic potential of a model combining EIPs for sepsis identification. Our primary logarithmic model demonstrated excellent discrimination in a retrospective case-control cohort. The high performance is biologically plausible, as it integrates markers of the innate immune response (IG# and NEUT-RI) and the adaptive immune response (RE-LYMP%/L). The internal validation results further suggest that the model is statistically stable, with minimal overfitting to the development data.

### 4.1 Cost-effectiveness considerations

The economic implications of EIP-based sepsis screening merit consideration:

#### 4.1.1 Initial investment

Implementing EIP-based sepsis screening may involve a one-time software activation license costing between $5,000 and $10,000 per analyzer. Notably, there are no additional reagent expenses, as EIP utilizes standard CBC reagents. Laboratory staff require only 2–4 h of training, making the onboarding process relatively quick and cost-effective.

#### 4.1.2 Per-test cost comparison

EIP markers incur a minimal incremental cost of < $0.50 per test, primarily covering electricity and maintenance. In contrast, traditional biomarkers are significantly more expensive: PCT tests range from $25 to $50, CRP tests from $10 to $20, and lactate tests from $15 to $25 per test.

#### 4.1.3 Time-to-result advantage

EIP testing, when combined with a standard CBC, delivers results within approximately 15 min of receiving the sample. This is considerably faster than PCT which takes 2–4 h, although newer point-of-care testing platforms can reduce this turnaround time to 40–60 min albeit at higher per-test costs. This is also faster than CRP (1–2 h), and blood cultures, which can take 24–72 h. The rapid turnaround of EIP testing offers a critical advantage in time-sensitive conditions like sepsis.

#### 4.1.4 Potential annual savings (500-bed hospital, 10,000 screens/year)

For a 500-bed hospital conducting 10,000 sepsis screenings annually, PCT-based screening could cost between $250,000 and $500,000. In contrast, EIP-based screening would cost around $5,000 annually after the initial investment, potentially saving $245,000 to $495,000 each year. Additionally, the 1.75–3.75-h faster turnaround time associated with EIP could reduce sepsis mortality by 7–30%, leading to fewer ICU days, shorter hospital stays, and significant cost reductions.

However, these promising findings must be interpreted with extreme caution due to the study’s significant methodological limitations.

### 4.2 Potential for severity stratification

Beyond the primary diagnostic question, our study noted a secondary signal regarding the model’s potential for severity stratification. As shown in Table 4, the EIP model score was significantly higher in patients with established septic shock (*n* = 38) compared to those with sepsis with organ dysfunction without shock (*n* = 15) 4, yielding a high apparent AUC of 0.912.

While intriguing, this finding must be interpreted with extreme caution as this analysis is cross-sectional and demonstrates correlation with established shock and may not predict the future progression. Secondly, the analysis is confounded by definition of septic shock by Sepsis-3 criteria, with a significantly higher baseline severity and our EIP model may simply be a proxy for this pre-existing, clinically apparent organ dysfunction. Third, this high apparent AUC, derived from a small subgroup of 53 patients, was not internally validated and is likely overfit. This secondary finding is purely hypothesis-generating for future prospective studies.

### 4.3 Comparison to established biomarkers and clinical scores

A critical limitation of our study is the absence of contemporaneous measurement of established sepsis biomarkers (PCT, CRP, lactate) and clinical scoring systems (qSOFA, NEWS2, SOFA) within the same patient cohort. This lack of head-to-head data collection, inherent to our retrospective design, precludes any direct comparison or definitive assessment of whether the EIP model provides incremental diagnostic value beyond these existing tools. Furthermore, due to the substantial inflation of our model’s performance metrics caused by severe spectrum bias (as discussed in section), any indirect comparison to published performance data for PCT, CRP, or clinical scores would be unreliable and potentially misleading. Therefore, we make no claims regarding the comparative performance of our model relative to current standards based on this study.

### 4.4 Roadmap for prospective validation

To address the inherent limitations of our exploratory case-control study, we are actively developing a rigorous prospective validation protocol. The planned study will adopt a single-gate prospective cohort design, enrolling consecutive adult patients presenting to the Emergency Department (ED) with suspected infection. Inclusion criteria will require the presence of ≥ 2 SIRS criteria alongside clinical suspicion of infection. We aim to enroll 1,000 patients, anticipating 120–180 sepsis cases based on an estimated prevalence of 12–18%.

The primary objective is to externally validate the three-parameter EIP model in terms of both discrimination and calibration within a clinically relevant ED population. Secondary objectives include a head-to-head comparison with established biomarkers sepsis biomarkers (PCT, CRP, lactate) and clinical scoring systems (qSOFA, NEWS2, SOFA), as well as decision curve analysis to evaluate net clinical benefit and an economic evaluation of model implementation.

Methodologically, the study will incorporate prospective data collection, blinded outcome adjudication by expert panels using Sepsis-3 criteria, and a prespecified statistical analysis plan to ensure robustness and reproducibility. This initiative represents a substantial 24–36 month commitment to comprehensive validation, and until its completion, our current findings should be considered hypothesis-generating rather than clinically actionable.

## 5 Limitations

### 5.1 Spectrum bias and its impact

Our case-control design’s spectrum bias warrants quantitative estimation of its effect on reported performance. Based on systematic reviews of diagnostic accuracy studies, two-gate designs comparing severely diseased patients to healthy controls typically inflate (12):

- AUC by 0.15–0.25 (adjusted estimate: 0.941 → 0.69–0.79 in ED populations)
- Sensitivity by 15–30% (adjusted estimate: 88.5% → 58–74%)
- Specificity by 10–25% (adjusted estimate: 91.3% → 66–81%)
- Positive likelihood ratios by 3–5 fold (adjusted estimate: 10.2 → 2.0–3.4).

Therefore, while our model demonstrates proof-of-principle biomarker integration, the reported AUC of 0.941 is not generalizable. The clinically realistic performance in Emergency Department patients with suspected sepsis (where sepsis must be distinguished from viral infections, non-infectious SIRS, and other inflammatory conditions) would likely show modest discrimination (AUC 0.70–0.79) based on literature estimates of spectrum bias effects (24), necessitating complete model redevelopment and recalibration in the target population before any clinical consideration. Also, the absence of contemporaneous measurement of established biomarkers (PCT, CRP, lactate) prevents assessment of incremental diagnostic value.

#### 5.1.1 Calibration

This study did not include prospective assessment of model calibration. Calibration, the agreement between predicted probabilities and observed outcomes, is essential for a model to be clinically useful, as decisions are often based on absolute risk thresholds. Indeed, our *post hoc* calibration analysis revealed poor calibration (Hosmer-Lemeshow *p* < 0.001, calibration-in-the-large = 2.80, calibration slope = –0.06), confirming that the model’s probability estimates are unreliable in this artificial case-control scenario.

#### 5.1.2 Timing of sample collection

These were the first blood samples collected at presentation, but there was a median delay of 6.2 h which limits applicability to early sepsis detection where rapid triage decisions are crucial.

#### 5.1.3 Selection bias and geographic generalizability

This single-center, retrospective study enrolled non-consecutive controls from outpatient clinics rather than the clinically relevant population of consecutive Emergency Department patients with suspected infection. This selection approach introduces multiple biases:

- Healthy volunteer bias: Outpatient controls were selected for absence of acute illness, creating artificial separation.
- Geographic limitation: Single institution in the United Arab Emirates may not reflect diverse global populations, pathogen profiles, or healthcare systems.
- Temporal bias: Retrospective data collection prevents standardized prospective protocols.

External validation across multiple centers, healthcare systems, and demographic populations is essential before any claims of generalizability can be considered.

#### 5.1.4 Platform dependence

The EIP measurements were derived from a single analyzer model (Sysmex XN-1000), and performance may vary across different platforms.

#### 5.1.5 Data-driven optimization

The scaling factors used in the model were determined through grid search on the same dataset, potentially introducing additional optimism.

#### 5.1.6 Absence of external validation

While internal bootstrap validation provides insight into the model’s stability within the development cohort, it cannot replace external validation across independent datasets. Our study lacks temporal validation (performance across different time periods), geographic validation (across institutions, countries, or healthcare systems), population validation (across diverse demographics, comorbidities, and pathogen profiles), and platform validation (across different hematology analyzer models or manufacturers). Without these forms of external validation, the generalizability and transportability of our findings to other clinical settings remain uncertain.

## 6 Conclusion

In conclusion, a model combining the EIPs with IG#, NEUT-RI, and RE-LYMP%/L demonstrates high discriminatory performance for sepsis in a highly selected, retrospective case-control population. While the model appears statistically stable upon internal validation, its performance is likely inflated due to severe spectrum bias. These results should be considered preliminary and hypothesis-generating only, with no immediate clinical applicability. The model requires complete redevelopment and validation in its intended use population before any consideration of clinical implementation. Future validation must also include head-to-head comparison with established biomarkers (PCT, CRP) to determine incremental clinical value and address the single-platform dependency through multi-center studies using diverse hematology analyzers.Rigorous, large-scale prospective validation in a single-gate cohort of undifferentiated patients with suspected infection is imperative to assess the true accuracy, calibration, and clinical utility of this model before it can be considered for any clinical application.

## Data Availability

The raw data supporting the conclusions of this article will be made available by the authors, without undue reservation.
